# Group Appointments in a Breast Cancer Survivorship Clinic

**Published:** 2013-11-01

**Authors:** Kathryn Trotter, Susan M. Schneider, Barbara S. Turner

**Affiliations:** From Duke University School of Nursing, Durham, North Carolina

Breast cancer survivors have unique needs after the active treatment period is completed. They may have to deal with long-term adverse effects of cancer and its treatment such as chronic fatigue, lymphedema, pain, diminished concentration, weight gain, limited mobility, and sexual dysfunction (Hewitt, Greenfield, & Stovall, 2006; Jacobs et al., 2009; Miller, 2008). Psychosocial morbidity—including anxiety, depression, changed body image, and personal relationships (Hart, 2007)—is an issue, as is the increased risk of recurrent malignancy and late effects such as cardiovascular and pulmonary disease (Ganz, 2009; Hollowell et al., 2010).

Patients are often discharged from active treatment without guidance, education (Ganz, 2009), or psychosocial support (Cappiello, Cunningham, Knobf, & Erdos, 2007). The surmised plan is that they will return to their previous lives and work toward regaining any deficits as soon as possible. Some cancer survivors are sufficiently empowered to ask medical providers what to expect or how to cope after completion of active treatment, but many are not (Corner, 2008).

At a comprehensive cancer center in the southeastern United States, the returning breast cancer survivor often has a wait of 1 to 4 hours in the clinic waiting room, sitting among newly diagnosed and active treatment patients who are there to see the same specialists. This experience has been described by many as emotionally difficult. The wait is followed by a brief session with the oncologist, focusing on cancer surveillance. Little time is available to discuss long-term adverse treatment effects, as the oncologists have difficulty finding appointment times for new patients. This trend reflects a national workforce projection that there are too few oncologists to serve the increasing number of cancer patients (Hewitt et al., 2006).

To help increase the availability of survivor services and relieve the schedules of two busy medical oncologists at this institution, a group medical appointment visit for breast cancer survivors was initiated (Trotter, Frazier, Hendricks, & Scarsella, 2011), adapted from the Centering Healthcare Institute model of group care (Rising, 1998). This model—which offers peer support, education, and assessment in one space—was seen as an ideal format for survivors in follow-up care. Group medical appointments, also called shared medical appointments, are designed to bring together several patients with a similar health issue. The participants share a facilitated group discussion in addition to their individual visits with a health-care provider.

In this particular execution of the model, referrals who were 3 years or more from diagnosis and free from metastatic disease were eligible to participate. Many were on endocrine therapy. Each group comprised six patients who were due for their follow-up visit and were scheduled in the same block appointment time. Having six survivors allows for strong group dynamics, avoids extended waiting times between services, and provides reimbursement income. The group of survivors may choose to return for the next visit as a cohort.

## Evidence Related to the Group Medical Appointment Model

Breast cancer survivors have unique needs after the active treatment period is completed. They may have to deal with long-term adverse effects of cancer and its treatment such as chronic fatigue, lymphedema, pain, diminished concentration, weight gain, limited mobility, and sexual dysfunction (Hewitt, Greenfield, & Stovall, 2006; Jacobs et al., 2009; Miller, 2008). Psychosocial morbidity—including anxiety, depression, changed body image, and personal relationships (Hart, 2007)—is an issue, as is the increased risk of recurrent malignancy and late effects such as cardiovascular and pulmonary disease (Ganz, 2009; Hollowell et al., 2010).

Patients are often discharged from active treatment without guidance, education (Ganz, 2009), or psychosocial support (Cappiello, Cunningham, Knobf, & Erdos, 2007). The surmised plan is that they will return to their previous lives and work toward regaining any deficits as soon as possible. Some cancer survivors are sufficiently empowered to ask medical providers what to expect or how to cope after completion of active treatment, but many are not (Corner, 2008).

At a comprehensive cancer center in the southeastern United States, the returning breast cancer survivor often has a wait of 1 to 4 hours in the clinic waiting room, sitting among newly diagnosed and active treatment patients who are there to see the same specialists. This experience has been described by many as emotionally difficult. The wait is followed by a brief session with the oncologist, focusing on cancer surveillance. Little time is available to discuss long-term adverse treatment effects, as the oncologists have difficulty finding appointment times for new patients. This trend reflects a national workforce projection that there are too few oncologists to serve the increasing number of cancer patients (Hewitt et al., 2006).

To help increase the availability of survivor services and relieve the schedules of two busy medical oncologists at this institution, a group medical appointment visit for breast cancer survivors was initiated (Trotter, Frazier, Hendricks, & Scarsella, 2011), adapted from the Centering Healthcare Institute model of group care (Rising, 1998). This model—which offers peer support, education, and assessment in one space—was seen as an ideal format for survivors in follow-up care. Group medical appointments, also called shared medical appointments, are designed to bring together several patients with a similar health issue. The participants share a facilitated group discussion in addition to their individual visits with a health-care provider.

In this particular execution of the model, referrals who were 3 years or more from diagnosis and free from metastatic disease were eligible to participate. Many were on endocrine therapy. Each group comprised six patients who were due for their follow-up visit and were scheduled in the same block appointment time. Having six survivors allows for strong group dynamics, avoids extended waiting times between services, and provides reimbursement income. The group of survivors may choose to return for the next visit as a cohort.

The group medical appointment model is a patient-centered, cost-effective care innovation that improves access, outcomes, and care quality (Martin et al., 2004; Noffsinger, 2008; Agency for Healthcare Research and Quality, 2009). It has been employed with chronic illnesses such as diabetes (Beck et al., 1997; Wagner et al., 2001; Loney-Hutchinson et al., 2009; Scott et al., 2004), heart failure (Yehle, Sands, Rhynders, & Newton, 2009; Lin, Cavendish, Boren, Ofstad, & Seidensticker, 2008), rheumatoid arthritis (Shojania & Ratzlaff, 2010), and asthma (Rhee, Ciurzynski, & Yoos, 2008) as well as in preventive care settings such as prenatal care (Ickovics et al., 2007), well-child care (Osborn & Woolley, 1981; Taylor, Davis, & Kemper, 1997), and cancer care (Trotter et al., 2011). Furthermore, it has been developed in rural and urban populations, both nationally and internationally, particularly for diabetes care (Bray et al., 2005; Clancy et al., 2008; Trento et al., 2010; Vachon et al., 2007).

This model—also referred to as shared medical appointments, group medical visits, group care, cooperative health-care clinics, and chronic care clinics—gives patients the opportunity to receive one-on-one medical assessment and patient education within a framework of social support from peers dealing with similar issues. Group visits are a vehicle to involve and empower patients. These visits can build confidence and self-management skills while encouraging individuals to set and meet appropriate goals (Barud, Marcy, Armor, Chonlahan, & Beach, 2006; Jaber, Braksmajer, & Trilling, 2006a, 2006b).

Practices have also reported improved efficiency in access to care, which assists with the financial impact on those practices (Sidorsky, Huang, & Dinulos, 2010). This is important in the current economy. Since 2005, the American Academy of Family Physicians (2011) has published practice redesign tools to assist family physicians to set up group visits. In addition, both the Institute for Healthcare Improvement (2011) and the Agency for Healthcare Research and Quality (2009) endorse the concept as an innovation that shows promise for quality care.

## Group Medical Appointment Structure

In the particular model discussed in this article, appointment times were respected, and team members promptly invited patients to the survivor clinic group space to meet and greet the clinicians and each other. The format included a 15-minute check-in period during which patients took their own vital signs and updated their treatment summary and care plan on an institution-specific document hand-generated by the nurse practitioner (NP) prior to the visit (see Appendix A1). This was followed by a 45-minute facilitated group discussion with the six survivors there for their follow-up visit.

Structured with initial completion of a self-assessment sheet (see Appendix B), the discussion often revolved around chronic issues such as menopausal symptoms, bone health, libido issues, insomnia, and the latest media information about cancer. An NP, a registered dietitian, a physical therapist, and a social worker were present for the sessions. Thereafter, the participants moved to their individual exams with the NP, but some first went (often in tandem, as an extension of the group camaraderie) for their mammogram and returned later for the exam. Between the examination and the mammogram, participants would spend time further discussing nutritional issues with the dietitian or stress management and relationship issues with the social worker.

Before exiting, the NP reviewed the individual treatment summary care plan with each patient. The NP completed a specific health-care plan on the form, including recommendations for various cancer screenings, while the patient wrote both her short- and long-term personal goals. This did not replace a more detailed customary chart note. This treatment summary and plan is consistent with both the Institute of Medicine (Hewitt, Greenfield, & Stovall, 2006) and the American Society of Clinical Oncology (Khatcheressian, 2012) recommendations to help improve documentation and coordination of cancer treatment and survivorship care.

It took most patients a total of approximately 2.5 hours to completely work through all of the services (group session; radiologist-reviewed mammogram; exam and medical care with the NP; and consult with the dietitian, physical therapist, and/or social worker). Billing was done using traditional evaluation and management coding, as there is not yet a recognized provider-attended group visit reimbursement code. If abnormal findings were noted, either on exam or imaging, the NP; further evaluated them and referred the patient back to the primary oncologist when indicated.

Anecdotally, patients seemed satisfied with this model; the majority of participants returned. Indeed, patient satisfaction has been recognized as one of the key indicators of health-care quality and is now being used by health-care institutions for monitoring health-care improvement programs, gaining accreditation, and developing marketing strategies (Mainz, 2003; Kleeberg et al., 2005). The patient satisfaction information is also being used to compare and benchmark hospitals (Coulter & Cleary, 2001), identify the best-performing institutions, and discover areas in need of improvement.

## Analysis of the Two Main Study Questions

A project analysis was necessary to answer the following questions, especially as the findings may impact future expansion of the program (hereafter referred to as Question 1 and Question 2): (1) Does a group visit cancer survivor care model that is introduced into a large cancer center result in high patient satisfaction? (2) Does the model provide a relevant cost benefit that fits within the strategic plan of the organization? To capture the impact of this model fully, these questions will first be examined separately and then reviewed together to understand the potential benefit to the organization.

## Methods

**Question 1:**Does a group visit cancer survivor care model that is introduced into a large cancer center result in high patient satisfaction?

Specifically, do the following variables make a difference in patient satisfaction: time since diagnosis, age at time of diagnosis, or time of day for clinic? Was the survivor summary treatment and care plan thought to be helpful? What did patients like the most and least about the program? 

This study offered patients an opportunity to evaluate their breast cancer survivor clinic (BCSC) visit. A 22-item program evaluation form was developed (see Appendix C). Faculty content experts reviewed the survey for validity, the readability score was 7.8, and a pilot established understandability and time to completion. Twenty Likert-type questions sought their feedback on the clinic process, clinician concern for their issues, development of their survivor care plan, and how likely they were to recommend this clinic to other survivors. The last two questions were open-ended, asking participants what they liked the most and least about the clinic.

From November 2010 to July 2011, following Institutional Review Board approval, clinicians offered the questionnaire as patients completed their group medical appointment. Following informed consent, the majority of patients returned the self-administered questionnaire before exiting the clinic. A preaddressed, stamped envelope was available to those patients who were in a hurry but wanted to participate by mail. Initially, patients utilized an electronic survey to complete the questionnaire (n = 12), but primarily a written two-page survey was completed, after informed consent was obtained.

**Question 2: ** Does the model provide a relevant cost benefit that fits within the strategic plan of the organization?

Specifically, what are the revenues and costs of the program? Was there a decrease in "time to third available appointment" for the referring provider(s)?

The cost-benefit analysis was a retrospective two-group design of clinic encounter financial data for follow-up breast cancer survivor patients. One group was the set of patients seen in the BCSC by the NP in fiscal year 2010 (n = 300), and the other group was a subset of patients seen by the referring medical oncologist for follow-up care during the same fiscal year (n = 300). Furthermore, to check for possible improvement in appointment availability, the "time to third available appointment," which is customarily followed by administration, was reviewed for the medical oncologist and the NP. Counting the third next available appointment is the health-care industry’s standard measure (Rose, Ross, & Horwitz, 2011) of access to care and indicates how long a patient waits to be seen.

## Results

**QUESTION 1**

Sample Size and Response Rate. Of the 167 patients seen in the clinic during the study, 122 surveys were completed (73% response rate). Of these, 22 of the surveys were incomplete, with one or more of the questions skipped. Where 1 or 2 question responses were missing, those items were not included in calculations for that particular question. Early in the study, not all patients were apparently offered the survey, which likely affected the response rate. This was remedied when all clinic team members were reminded of the survey activity, when the electronic method was abandoned due to connection complications, and after surveys were set out on clipboards prior to clinic start for ease of use and increased visibility. Thereafter, almost all patients submitted the survey.

Demographics. Providing a medical record number was optional for patients, and 70 (56%) did so. Of these, the mean years postdiagnosis was 11, with a range of 3 to 21 years. The average age of the participants was 64.3 years, with a range of 39 to 80 years. During the study time frame, the clinic provided follow-up care to the nonmetastatic breast cancer patients who were referred at 3 years or more postdiagnosis. The majority of patients had a stage I or II breast cancer history (see Table 1). This closely compares to the demographics of the entire clinic.

**Table 1:**
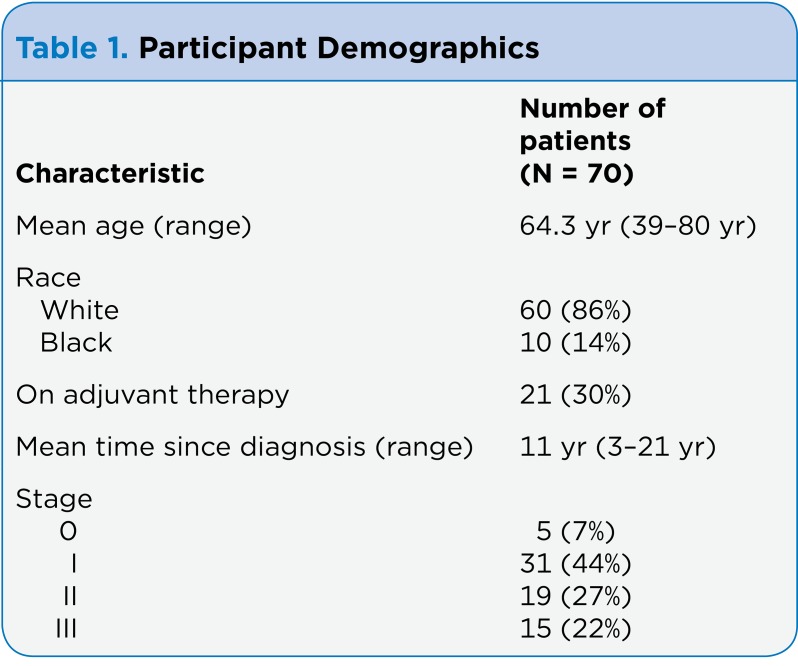
Table 1. Participant Demographics

Data Analysis. After review of data spreadsheets by two reviewers for accuracy, the analysis was completed using IBM’s SPSS v19. Descriptive statistics results are seen in Appendix C. Of note, the survey tool had a Cronbach’s alpha score of .90, supporting internal reliability of survey questions utilized.

The data show high scores for patient satisfaction overall, with mean scores ranging from 4.3 to 4.9 of 5 on a 0 to 5 scale, with 5 representing "strongly agree." The lowest score noted was related to mammography service (length of wait, question 4), and the highest score was for the two questions relating to confidence in the NP and the concern shown by the NP (4.93 and 4.93, respectively, questions 7 and 8).

For the purpose of this summary of data, the "strongly agree" and "agree" data were collapsed to indicate which percentage of patients agreed with the question. Almost all of the 118 respondents to question 12 (98%) "strongly agreed" or "agreed" that they liked the survivor clinic appointment done in this way (group session, then individual visit), and 97% of the 117 respondents to question 20 "strongly agreed" or "agreed" that they were likely to recommend the program to other breast cancer survivors. A full 100% of the 120 respondents to question 6 "strongly agreed" or "agreed" that the length of time spent with the NP was adequate, and 98% of the 117 respondents to question 19 "strongly agreed" or "agreed" that the program provided quality care.

Using analysis of variance, neither a patient’s age at diagnosis (less than 50 years old vs. 50 or older) nor the number of years since diagnosis (less than 10 years vs. 10 years or more) showed a significant difference in their mean scores (see Tables 2 and 3), nor did the time of day for the clinic (Thursday afternoon vs. Friday morning) demonstrate a significant difference in opinion. This was reassuring as the clinicians had sensed an eagerness to finish the visit and travel home with the afternoon sessions that was thought to relate to both the time of day and a scheduling miscue wherein the total wait time was longer for these sessions. The design for clinic flow usually features a decreased wait time, brought about by setting up half of the patients for their imaging service before the group session, and the other half after the group session. Using a one-way ANOVA test, the results demonstrated a slightly lower score for Thursday vs. Friday clinic (4.4 vs. 4.6, N = 104;* p* = .208), but it was not statistically significant.

**Table 2:**
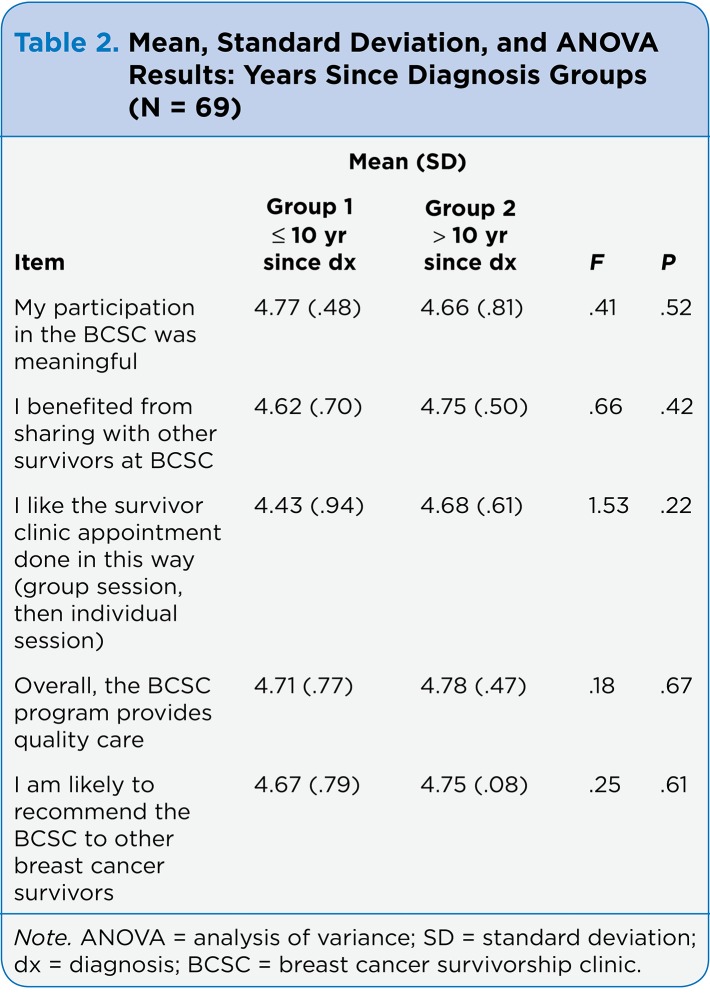
Table 2. Mean, Standard Deviation, and ANOVA Results: Years Since Diagnosis Groups (N = 69)

**Table 3:**
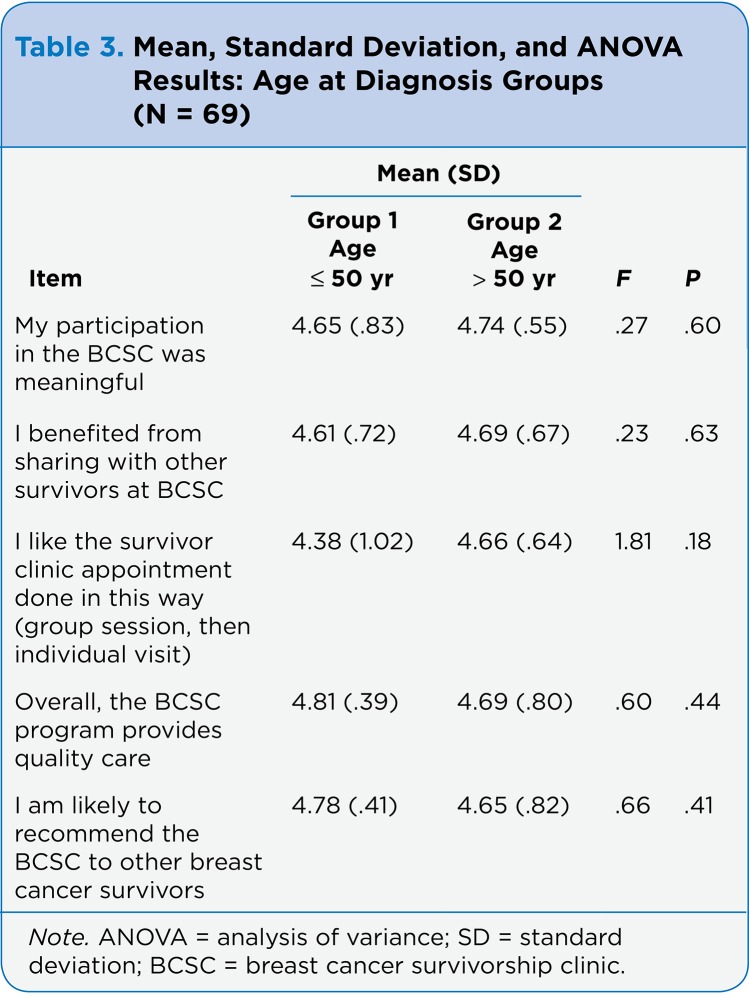
Table 3. Mean, Standard Deviation, and ANOVA Results: Age at Diagnosis Groups (N = 69)

Nearly all (93.4%) of the 121 patients answering question 15 "strongly agreed" or "agreed" that developing the care plan was helpful to them. However, 80.7% of the 114 respondents to question 16 "strongly agreed" or "agreed" that they plan to share the care plan with other health-care providers (such as their primary care provider or gynecologist). This was likely the first time in their cancer care that they had received a treatment summary and care plan, so it may have been a new idea to most of them.

Qualitative Analysis. All written responses were grouped by frequently written words or phrases, and themes were then identified. One other reviewer also evaluated the phrases and grouped them into themes. The interrater reliability was 90%. Over 80% of respondents added comments and the vast majority were positive. Three themes were noted concerning the most liked aspects of the clinic: (1) the sharing and camaraderie with other survivors; (2) health information (education) received, including updates or research about health topics; and (3) the caring, attentive professionalism of the staff. Of the 20% who commented about least liked aspects of the clinic, the wait in mammography area was the most frequently cited issue. Service issues such as appointment communication and group visit style or length were rarely mentioned (see Table 4).

**Table 4:**
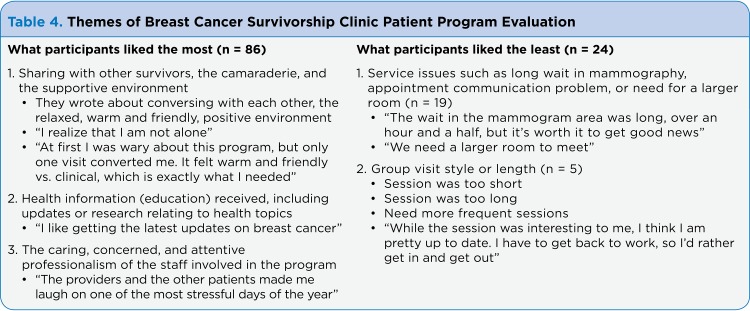
Table 4. Themes of Breast Cancer Survivorship Clinic Patient Program Evaluation

**QUESTION 2**

Cost-Benefit Analysis. Key assumptions included the following: This analysis was done from the institution’s perspective, not the patient or payer perspective; the no-show rate/cancellation rates were the same; and the clinic space, utility cost, and ancillary support away from the clinics were the same. It was determined that if less than 7.5% of the staff time was required for the BCSC clinic, their time with other department duties would not need to be replaced. An opportunity cost was negligible, as the group space had previously been unoccupied.

A revenue and cost comparison revealed that revenues were nearly equal between the delivery models. The cost of group visits was very slightly higher given the multidisciplinary team members who are not a billable service (dietitian and social worker). The physical therapist could bill if an evaluation was accomplished, but only about 10% needed this during the visit as they were at least 3 years postdiagnosis in their survivorship phase and had already addressed their lymphedema or range-of-motion issues. For this analysis, based on clinical observation, it was assumed that the NP spent twice the time per patient visit than did the physician, given the time spent with patients in the group session. Using highest cost estimates, the annual relevant cost of providing the clinic is $1,396, or $4.65 per patient per year (see Appendix D).

The review of "time to third available appointment" for new patients for the primary referring oncologist was measurable. It dropped from 29.4 days (in fiscal year 2009) to 26.7 days (in fiscal year 2010), whereas the NP time remained stable at 8.7 days.

## Discussion

Similar to other cancer program surveys, we found the patients to be highly satisfied with their care. Many studies evaluating patient satisfaction show positive results when asked about their health care, including the group visit format (Noffsinger, 2008; Ickovics et al., 2007). Sitzia (1999) noted that research instruments might lack reliability and validity assessment, which casts doubt on the credibility of satisfaction findings. Therefore, it is important to know that experts on content validity reviewed this instrument, and a pilot test was done with patients for readability and flow. There remains concern about whether these results are generalizable to other survivors who were not referred or the few who elected not to participate in this delivery model. Additionally, the model was implemented at one site, with a homogeneous population. A comparison group was not studied, and this would be recommended if repeated. However, this survey provided specific feedback on the process and flow of services and had a very high response rate.

This report showed that the number of years postdiagnosis made no difference in scores for "benefit from sharing with other survivors" or scores for "participation in the program was meaningful." This is reflected in the literature that indicates that female cancer patients may identify themselves as cancer survivors longer than male survivors. It has been postulated that this is a group whose members have a need to share their experiences and receive social support (Lee, 1997), and therefore participation in the group visit model may be well suited to these patients.

The design also included the immediate accessibility of a registered dietitian, social worker, and a physical therapist, which is not traditionally offered during a follow-up medical appointment. At this facility, it often takes 3 or more weeks to get an appointment with a physical therapist that specializes in lymphedema or women’s health issues. The score on convenience of services was 4.6, which shows good satisfaction. Another factor that could affect this score is the close proximity of x-ray and lab services and the fact that sessions start and end on time.

The total time with the NP was noted to be adequate by 98% of the respondents. This could be due to the high visibility of the NP who was present for both the 45- minute group session and the individual examination. Interestingly, the NP reported that the individual meeting time was slightly shorter and more efficient than traditional clinic visits that do not have the interactive discussion. Perhaps the time in the group visit allowed patients to ask questions while allowing the NP a chance to gather information about each patient. A time study should be coordinated to validate this point.

The financial computation was nearly equal in revenue, but expenses for salary coverage (for the lead administrative and clinical NP as well as the team clinicians) caused a slight net loss. Recently, the physical therapy department had not authorized the physical therapy specialist to come to the clinic due to high demand in her regular clinic. Further analysis of clinician consultation rates could help determine whether the dietitian, social worker, or physical therapist should remain for the post group session or could return to other duties. Clinic staff agreed that the dietitian was consulted the most, so perhaps the others could be available by pager afterward. This may be important when expansion of the clinic occurs.

Budgets for clinical operations are important baseline data, but reviewing other justifications for a program is also very important. The primary financial incentive for providing a separate survivorship clinic is that offloading the oncologist allows more patient consultations. Freeing up the oncologist’s schedule for newly diagnosed cancer patients should offer revenue to both the cancer center, which will administer and be reimbursed for cancer treatments, as well as to the oncologist, who will maintain a highly productive schedule. Measuring this schedule shift proved slightly difficult, but the oncology practice management administrator and the primary referring oncologist agreed that with a shift of three follow-up patients, an opening for one new patient could occur. With the average net contribution margin impact estimated at $8,400 per new patient, this survivor clinic is viewed as very cost beneficial to the organization.

Furthermore, the administrator suggested that the value of a survivorship clinic to the community image of the cancer center should not be underestimated. Indeed, these patients overwhelmingly agreed that they would refer other cancer survivors and likely newly diagnosed family and friends. It is difficult to assign a monetary value to this factor, but it was thought that new patients would likely choose a cancer center based on services available for total care, including survivorship services. Goldman and Chang (2010) report similar thoughts and point out that offering a service with intangible values that connects to the community, and to women in particular, is valuable to a cancer program.

## Conclusion

The model of survivorship care delivery described in this article is feasible and well received by patients. It is congruent with the Institute of Medicine (IOM) report on survivor care (Hewitt et al., 2006), which suggests recognition of cancer survivorship as a distinct phase of cancer care. The report stresses coordination, along with attention to survivor concerns, as a key issue in improving follow-up care. More experimentation, adaptation, and evaluation of survivorship clinic models are needed. Certainly, this clinic structure provides for psychosocial support, health promotion activities, survivor empowerment, and surveillance for tumor recurrence and late effects. It also features the creation of an individualized, written breast cancer survivorship care plan for each survivor to share with her primary care provider. Adaptation of the model is possible with attention to the essential elements of the Centering Healthcare Institute group care model.

Answering the financial question of whether benefits outweigh costs proved equivocal until the potential downstream revenue from newly diagnosed cancer patients was considered. The nonfinancial benefit of offering a survivorship program that supports an organization’s mission, its patients, and the community is important to consider. When future expansion is considered, looking at a product margin paradigm to make this budget decision will be key.

Attention to survivor needs and concerns with immediately available specialized clinicians can only improve the quality of care. Given this population’s resounding positive feedback on the group medical appointment format, this model of delivery should be considered on a larger scale, potentially with patients who are earlier in their survivorship phase and perhaps in other cancer survivorship arenas.

## ACKNOWLEDGMENTS

The authors would like to thank James R. Vroom, DHA, MHA, for his assistance with financial analysis; P. Kelly Marcom, MD, for his clinical support; and the survivors who actively participated in the group visits discussed in this article.
